# TASK channels in arterial chemoreceptors and their role in oxygen and acid sensing

**DOI:** 10.1007/s00424-015-1689-1

**Published:** 2015-01-28

**Authors:** Keith J. Buckler

**Affiliations:** Department of Physiology Anatomy and Genetics, University of Oxford, Parks Rd, Oxford, OX1 3PT England

**Keywords:** K2P channel, Potassium channel, TASK channel, Carotid body, Hypoxia, Oxygen sensing

## Abstract

Arterial chemoreceptors play a vital role in cardiorespiratory control by providing the brain with information regarding blood oxygen, carbon dioxide, and pH. The main chemoreceptor, the carotid body, is composed of sensory (type 1) cells which respond to hypoxia or acidosis with a depolarising receptor potential which in turn activates voltage-gated calcium entry, neurosecretion and excitation of adjacent afferent nerves. The receptor potential is generated by inhibition of Twik-related acid-sensitive K^+^ channel 1 and 3 (TASK1/TASK3) heterodimeric channels which normally maintain the cells’ resting membrane potential. These channels are thought to be directly inhibited by acidosis. Oxygen sensitivity, however, probably derives from a metabolic signalling pathway. The carotid body, isolated type 1 cells, and all forms of TASK channel found in the type 1 cell, are highly sensitive to inhibitors of mitochondrial metabolism. Moreover, type1 cell TASK channels are activated by millimolar levels of MgATP. In addition to their role in the transduction of chemostimuli, type 1 cell TASK channels have also been implicated in the modulation of chemoreceptor function by a number of neurocrine/paracrine signalling molecules including adenosine, GABA, and serotonin. They may also be instrumental in mediating the depression of the acute hypoxic ventilatory response that occurs with some general anaesthetics. Modulation of TASK channel activity is therefore a key mechanism by which the excitability of chemoreceptors can be controlled. This is not only of physiological importance but may also offer a therapeutic strategy for the treatment of cardiorespiratory disorders that are associated with chemoreceptor dysfunction.

## Arterial chemoreceptors: background to structure and function

Arterial chemoreceptors are among the smallest organs in our bodies. Their function is to serve both cardiovascular and respiratory control centres in the brainstem with vital information concerning the chemical composition of arterial blood. There are two main clusters of chemoreceptor tissue, that in the vicinity of the common carotid artery, which normally forms well-defined bilateral organs called carotid bodies, and that associated with the underside of the aortic arch (and sometimes in the vicinity of the pulmonary and subclavian arteries), which form less well-defined aggregates of tissue called aortic bodies. Of these, it is the carotid bodies which probably have the most important physiological role and which have been the most extensively studied. The carotid body is composed of small clusters of type 1 (glomus) cells partially enveloped by glial-like type 2 (sustentacular) cells. Type 1 cells are innervated by afferent neurons from the carotid sinus nerve which originate in the petrosal ganglion and project to the nucleus tractus solitarii. The carotid body is richly vascularised and is believed to have the highest resting blood flow per unit weight of any organ in the body (for further background to these organs, the reader is directed to a number of extensive reviews [31, 41, 45]).

The carotid body has a number of sensory modalities. Perhaps the most striking is the ability to sense changes in oxygen levels in the blood. The response of an isolated carotid body or chemoreceptor cell to a fall in oxygen levels is manifested within seconds. Acute oxygen sensing of this form is only recognised in a few other tissues, i.e. pulmonary arterioles, neuroepithelial bodies of the lung and neonatal adrenal medullary chromaffin cells [135]. The carotid body is also sensitive to acidosis and CO_2_ (probably via changes in pH_i_, see [41]). In addition to natural stimuli, the carotid body is also notable for being excited by mitochondrial poisons [3, 48, 87, 117] which may reflect a metabolic sensing capacity. In recent years, the carotid body has also been proposed to act as a blood glucose sensor [97, 144]. This is a controversial area with different groups and experimental approaches providing conflicting evidence [5, 25, 43]. It is possible that glucose sensitivity may again derive from some form of metabolic sensing pathway that is dependent upon both extracellular glucose availability and glycogen reserves [49].

Chemotransduction mechanisms are best described for oxygen sensing and acid/CO_2_ sensing. In both cases, the basic mechanism is a classical sequence of electrical excitation of the primary sensory cell, the type 1 cell, which leads to an influx of calcium and neurosecretion [13–15]. The type 1 cell contains a rich diversity of neurotransmitters which fulfil a variety of roles [41, 45, 89]. For the sake of brevity, however, current evidence suggests that ATP is probably the main excitatory transmitter released from the type 1 cell. This excites afferent nerve endings via P2X2/P2X3 receptors [114, 147, 148].

## Role of background K-currents and other channels in mediating electrical signalling

From the above description, it is clear that electrical signalling plays a central role in the transduction of chemostimuli. The basic model of electrical signalling is that hypoxia and acidosis modulate specific ion channels in the type 1 cell to elicit a depolarising receptor potential which is often accompanied by spontaneous action potentials. These electrical events incorporate the activation of voltage-gated calcium channels which mediate a large influx of calcium ions resulting in a substantive elevation of the intracellular calcium ion concentration [8, 14, 15]. Type 1 cells express a number of different ion channels including Twik-related acid-sensitive K^+^ channels (TASK channels)^+^, an uncharacterised background Na^+^ conductance, Cl^−^ channels, a calcium-activated cation channel, L- and N-type voltage-gated calcium channels, voltage-gated Na^+^ channels (in some species), voltage-gated (delayed rectifier) K^+^ channels and large conductance calcium-activated K^+^ channels (maxi K channels, BK_Ca_) [8, 17, 19–21, 35, 38, 53, 72, 74, 101, 104, 113, 120, 121, 130]. The functional role of all of these channels however is not necessarily well understood.

The resting potential of acutely isolated type 1 cells in vitro in physiological media at 35–37 C as recorded using the perforated patch technique is around −50 to −60 mV [9, 14, 15, 113, 139]. As in other cells, this resting potential represents a balance between inward currents, most likely carried by Na^+^ ions, and outward currents carried by K^+^ ions and the Na^+^/K^+^ pump. The resting (background) K^+^ conductance of the type 1 cell was estimated at around 340 pS [8] which, at a membrane potential of −50 mV, would generate an outward K^+^ current of around 14 pA. This resting potassium conductance is largely voltage insensitive and appears to be mediated primarily by TASK channels [17]. The channels responsible for resting Na^+^ ion influx have not yet been identified although preliminary estimates suggest that this is not an insignificant current (approx. −20 pA) [8, 20]. In the steady state, the corresponding Na^+^ influx would have to be balanced by Na^+^/K^+^ pump activity which, with a coupling ratio of 3Na^+^/2 K^+^, would generate an additional outward current of about 7 pA. Thus, the charge movements through these three pathways are roughly in balance. It is probable that other resting membrane currents also exist, we know little about Cl^−^ currents in type 1 cells for instance.

The resting background K^+^ conductance is inhibited in response to hypoxia and acidosis [8, 15, 17]. Under typical experimental hypoxic conditions, the background K^+^ conductance may be reduced by between 50 and 80 % [17, 56]. A 50 % reduction in background K conductance would result in a decline in the outward resting K-current from 14 to 7 pA, thus creating an imbalance between resting inward and outward currents. The resultant net inward current (−7 pA), carried predominantly by Na ions, will then bring about a rapid membrane depolarisation. Thus, the initial membrane depolarisation is primarily a consequence of background (TASK) K^+^ channel inhibition but is probably driven by Na^+^ ion influx.

**Fig. 1:**
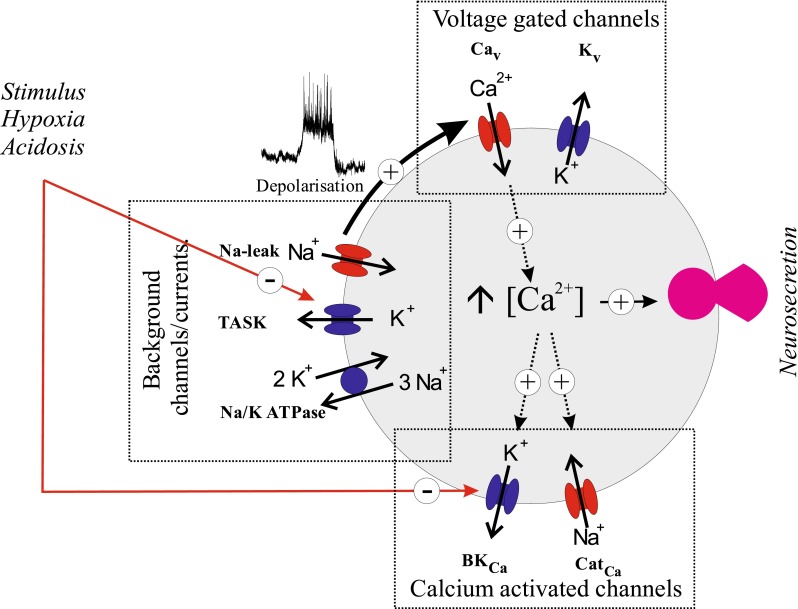
Ion channels and electrical signalling in type 1 cells. Putative summary model of key ion channels/currents in rat type 1 cells. Background channels and currents setting the resting potential include **TASK**, predominantly TASK1/TASK3 heterodimers plus some contribution from TASK1 and TASK3 homodimers; **Na-leak**, an uncharacterised background Na^+^-leak conductance; and **Na/K ATPase**, an Na/K pump current presumed to be present in order to maintain intracellular Na^+^ homeostasis. Voltage-gated channels mediating electrical activity (action potentials) in the rat type 1 cell include **Ca**_**V**_, voltage-gated calcium channels including L-type and N-type channels; **K**_**V**_, voltage-gated, delayed rectifier type, potassium channels (other species may also have voltage gated Na^+^ channels). Calcium-activated channels include **BK**_**Ca**_, a large conductance calcium-activated potassium channel (note this channel is also voltage sensitive) and **Cat**_**Ca**_, a calcium-activated cation channel permeable to Na^+^ ions. Signalling pathway for both hypoxia and acidosis involves inhibition of TASK channels (all three forms) and maxi-K^+^ channels. Inhibition of TASK leads to membrane depolarisation, followed by activation of voltage-gated Ca^+^ channels generating Ca^2+^ influx and upstroke of action potentials; K_V_ is assumed to mediate action potential repolarisation. The resulting rise in [Ca^2+^]_i_ not only promotes neurosecretion but also activates a non-selective cation channel which reinforces the depolarising effect of TASK channel inhibition. Ca^2+^-dependent activation of maxi-K channels would be expected to repolarise the type 1 cell and limit Ca^2+^ influx were it not also inhibited by hypoxia/acidosis [102, 103]. Channels in *red* mediate inward (depolarising) current, those in *blue* mediate outward (repolarising/hyperpolarising) current

Whilst we can account for the onset of membrane depolarisation in response to hypoxia through TASK channel inhibition, we do not yet know what defines the magnitude of the resulting receptor potential and any consequential electrical activity. A number of other channels are likely to play an increasingly significant role as the cell depolarises. Of particular relevance are high threshold voltage-gated Ca^2+^ channels which become active at potentials positive to −50 mV [15, 38, 130] and voltage-gated Na^+^ channels (in those species whose type 1 cells express them, e.g. rabbit [35]). This would add a further drive to depolarisation with positive feedback between depolarisation and Ca^2+^ and Na^+^ channel activation resulting in an action potential. In addition, a calcium-activated cation channel has recently been found in the type 1 cell. This channel becomes active during hypoxia due to the elevation of intracellular calcium (via voltage-gated Ca channels) and would thus contribute to a positive feedback driving further membrane depolarisation and Ca influx [53]. Counterbalancing these inward currents, we would expect depolarisation to activate voltage-gated K channels and elevation of cytosolic calcium to activate calcium-activated potassium channels both of which would generate outward repolarising current (and thus attenuate Ca influx). It is notable in this context that the large conductance calcium-activated potassium channels (maxi-K channels) in type 1 cells are also inhibited by hypoxia and acidosis [101, 102]. Inhibition of maxi-K channels may therefore facilitate electrical and calcium signalling in response to physiological stimuli. The exact role of these channels in controlling cellular electrical activity and calcium influx during excitation however requires further investigation. There may also be other chemosensitive currents present, e.g. acid-sensing ion channels (ASICs) can be activated under severe acidic conditions and may contribute a very brief depolarising current [126] in response to a rapid fall in pH. In addition, there may be other oxygen-sensitive currents present in type 1 cells [129]. Figure 1 shows a schematic of the main ion channels present in rat type 1 cells and their proposed role in electrical signalling.

## Calcium signalling in type 1 cells

Calcium influx and elevation of calcium concentration in the type 1 cell is thought to be a prerequisite for stimulus evoked neurosecretion [18, 106, 108]. The mechanisms of intracellular calcium regulation in type 1 cells have however received relatively little attention. The primary cause of the rise in [Ca^2+^]_i_ in response to hypoxia or acidosis is calcium influx through voltage-gated channels as described above [14, 15]. Calcium induced calcium release via ryanodine receptors is thought to be of minor significance since caffeine releasable stores seem small in type 1 cells [15]. Type 1 cells do however have intracellular calcium stores from which Ca^2+^ release may be evoked by receptor-activated signalling pathways (e.g. muscarinic receptors [32]), and it is possible that these have an important role to play in calcium signalling in vivo where the type 1 cell will be exposed to a variety of autocrine, paracrine and neurocrine factors [89]. Na^+^/Ca^2+^ exchange seems to have minimal role in mediating either calcium influx or efflux in type 1 cells [15], so we presume that the primary mechanism of Ca^2+^ extrusion from the cell is by a plasma membrane Ca^2+^/H^+^ ATPase although this remains to be confirmed. The role of mitochondria in calcium buffering has also not yet been studied, but given the exceptional sensitivity of mitochondrial membrane potential to hypoxia, it is possible that mitochondrial calcium uptake may be constrained in hypoxia which could facilitate Ca^2+^ signalling.

## Molecular and biophysical properties of TASK channels in type 1 cells

Notwithstanding the importance of numerous channels, pumps and receptors involved in type 1 cell signalling; it is clear that TASK channels play a key role in initiating the receptor potential in response to chemostimuli. This event is pivotal to the chemotransduction process.

It was the acid sensitivity of the background K^+^ current in type 1 cells, amongst other features, that led to the initial identification of this current as being TASK-like [17]. Other TASK-like features include little voltage sensitivity, other than that which may be ascribed to Goldman-Hodgkin-Katz type rectification, and a resistance to the classical K channel blockers TEA and 4-AP [8, 17]. Cell attached patch recordings from type 1 cells show abundant potassium channel activity at the resting potential. These channels have fast flickery kinetics with short bursts of rapid openings with estimated mean open time constant of 0.3 ms, a short closed time constant of 0.1 ms and a burst duration of 1.7 ms [137]. Long closed times are more difficult to measure as most patches contain a number of channels. Kinetic analysis of the cleanest recordings from patches with relatively low channel activity suggest the existence of only one long closed state but with a highly variable closed time of between 2 and 30 ms [137]. The mean single channel conductance was initially estimated at about 14 pS [17], but it was subsequently discovered that this is dependent upon extracellular magnesium. Early measurements were made presence of 4 mM extracellular (pipette) Mg^2+^. In the absence of extracellular Mg^2+^, mean single channel amplitude is about 28 pS [137]. Whilst these biophysical properties were broadly consistent with a TASK-like channel, it was notable that single channel conductance, and its dependence upon extracellular Mg^2+^, was distinct from that of both TASK1 and TASK3. This led to the suggestion that the native TASK-like channel might be a heterodimer [137] as TASK1 and TASK3 had been reported to heterodimerise in some expression systems [28].

Subsequent analysis of single channel records in rat type 1 cells, with particular emphasis on single channel conductance and its sensitivity to extracellular magnesium, led Kim et al. to propose that type 1 cells contain a mixture of homomeric TASK1 and TASK3 channels together with a third channel type that was intermediate in size having a conductance equivalent to that of a TASK1-TASK3 concatameric channel [56]. It was proposed that this third, and most abundant, form of channel activity arose from a native heterodimeric assembly of both TASK1 and TASK3 subunits [56]. Further studies in transgenic mice have confirmed that the predominant form of channel activity in type 1 cells is indeed a TASK1/TASK3 heterodimer [129]. In type 1 cells from wild-type mice, the principal form of single channel activity in cell-attached patches was found to have a single channel conductance of 33 pS (in 1 mM Mg^2+^). This channel was absent in type 1 cells obtained from both *Task1* knockout and *Task3* knockout mice, indicating that both genes are necessary for the formation of the wild-type channel. In its place however was observed a larger conductance TASK3-like channel in *Task1* ^*−/−*^ mice and a smaller conductance TASK1-like channel in *Task3* ^*−/−*^ mice. Neither of these channels nor the wild-type channel were observed in type 1 cells from double-knockout mice (*Task1* ^*−/−*^ */Task3* ^*−/−*^) nor indeed were TASK1-like channels seen in *Task1* ^*−/−*^ cells or TASK3-like channels seen in *Task3* ^*−/−*^ cells, thus confirming that the channels present in *Task1* ^*−/−*^ and *Task3* ^*−/−*^ were indeed homomeric TASK3 and TASK1, respectively [129].

In a small number of patches from normal mouse type 1 cells, a TASK1-like channel was also observed, suggesting that both homomeric TASK1 channels and heteromeric TASK1/TASK3 are present in mouse type 1 cells as is observed in rat type 1 cells [56, 129]. The main form of channel activity present however is the TASK1/TASK3 heteromultimer (in both species) which suggests that heterodimerisation is preferred to homodimerisation. Indeed, it was noted that the prevalence of heteromeric TASK1/TASK3 channels in normal type 1 cells was much greater than that of the homomeric TASK1 and TASK3 forms seen in the single knockout animals [129]. This suggests that heteromeric channels might be more readily formed or trafficked to the membrane, or more stable, or more active than homomeric channels.

## Consequences of constitutive genetic deletion of *Task1* and *Task3*

Deletion/knockout (KO) of *Task3* in mice has been reported to have no effect upon the ventilatory response to hypoxia. In contrast, deletion of *Task1* blunted the ventilatory response to hypoxia and depressed the chemoafferent (carotid sinus nerve) response to hypoxia in vitro [127]. Double knockout (DKO) of both *Task1* and *Task3* similarly reduced both ventilatory and chemoreceptor nerve responses to hypoxia but did not completely abolish them [127]. In studies conducted on type 1 cells in vitro, however, constitutive deletion of *Task3* and/or *Task1* appeared to have little effect upon either calcium signalling or neurosecretion in response to hypoxia (a small increase in basal neurosecretion was noted in the DKO) [17, 92].

Given the importance of TASK1/TASK3 in setting resting membrane potential, the loss of either or both of *Task1* and *Task3* would be expected to result in a sustained depolarisation of type 1 cells and an increase in resting [Ca^2+^]_i_. Resting [Ca^2+^]_i_ was however similar in all knockout animals [17], and resting membrane potential was only slightly depolarised in DKO (*Task1* ^*−/−*^ *;Task3* ^*−/−*^) mice [92]. This indicates that the loss of *Task1* and/or *Task3* must be largely compensated for in some way. Since K^+^ channel activity at negative membrane potentials was much reduced in all of these knockouts [17], and resting membrane conductance was much reduced in the DKO [92], the upregulation of another type of background potassium channel would seem not to be the principal means of compensation. Other possibilities include the downregulation of voltage-gated Ca^2+^ currents [92] which might help stabilise resting membrane potential and/or changes in the magnitude of other background currents, e.g. the inward (leak) Na^+^ current.

Irrespective of the means by which type 1 cells from KO and DKO animals avoid sustained depolarisation, what is more interesting is that they retain chemosensitivity to hypoxia. In single KO animals, this can be explained by the fact that the homomeric forms of TASK3 and TASK1 are also oxygen sensitive. In the case of the double knockout, however, it is clear that neither TASK1 nor TASK3 can be involved. Calcium-activated potassium channels (BK_Ca_) and some voltage-activated potassium channels (K_V_) also display oxygen sensitivity in type 1 cells [73, 74, 102, 104, 107, 143], but they tend to be already closed at potentials negative to −30 mV [92, 107]. BK_Ca_ and/or K_V_ channels would thus seem unlikely to be able to account for excitation of DKO type 1 cells. The implication of this data therefore is that type 1 cells must possess other, as yet unknown, oxygen-sensitive ion channels. *Task1* ^*−/−*^ *;Task3* ^*−/−*^ mice could therefore be a useful tool to aid the discovery of novel oxygen-sensitive channels. It should be noted however that the use of constitutive knockouts to infer the functional role of specific ion channels in normal animals is likely to be fraught with uncertainty. Naturally occurring variations in gene transcription from cell to cell combined with a mutation that has the potential to affect cell viability could well lead to the selective survival of an atypical cell population.

## Regulation of type 1 cell TASK channels by acidosis

Peripheral chemoreceptors are important sensors of blood acid base chemistry. Whilst they may contribute little more than 20–30 % of the respiratory drive in response to hypercapnic acidosis, the rest being mediated by central chemoreceptors, they are able to respond to an acute hypercapnia more rapidly than the central chemoreceptors. They are also able to detect metabolic acidosis (a fall in blood pH at constant CO_2_, e.g. as in diabetic ketoacidosis) which central chemoreceptors cannot. They can therefore provide a respiratory compensation for metabolic acid base disturbances. The transduction of acid stimuli, both hypercapnic and metabolic acidosis, involves the inhibition of background (TASK1/3) K^+^ currents much as for hypoxia [15, 17]. These channels are so named for their acid sensitivity with TASK1 being slightly more sensitive within the physiological range (*pK* = 7.2–7.3) than TASK3 (*pK* = 6.0–6.7) [36, 54, 55, 69, 79, 112]. The acid sensitivity of TASK1 and TASK3 is largely attributable to the protonation of a histidine residue in the large extracellular loop/helical cap region [55, 71, 86, 112]. Outside-out patch recordings of TASK-like channels in type 1 cells also show an external pH sensitivity with channel inhibition at pH less than 7.3 [56].

There is also evidence that the carotid body and type 1 cells can be excited by isohydric hypercapnia [13, 46, 65, 147]. Since this stimulus does not present an external acidosis, there must be another sensory mechanism probably linked to the intracellular acidosis caused by CO_2_ influx. This could be a direct effect of internal pH on TASK channels, or it might involve the metabolic signalling pathway (see below) since phosphofructokinase, a key enzyme and control point in glycolysis, is highly pH sensitive [128]. The effects of intracellular acidosis on TASK1/3 in these cells remain to be determined.

## Effects of hypoxia on TASK channels in type 1 cells

As noted above, the primary effect of hypoxia is to reduce background K^+^ currents in the type 1 cell, thus evoking membrane depolarisation, neurosecretion and excitation of the carotid sinus nerve. Studies conducted at the single channel level suggest that hypoxia reduces channel open probability mainly by stabilising the long closed state [17, 137]. Studies in knockout mice have further demonstrated that hypoxia can inhibit all three forms of these channels, i.e. TASK1/TASK3 heterodimers as well as homodimers of both TASK1 and TASK3 [129]. Although the effects of hypoxia on TASK3 appeared to be less dramatic than its effects on TASK1 and TASK1/TASK3, this data nonetheless indicates that the oxygen sensing pathway, whatever it may be, can couple to either TASK channel subunit.

Oxygen sensitivity in both TASK1 and the related channel TREK-1 has also been reported in heterologous expression systems (HEK293) [67, 81]. We however have never been able to replicate these findings [10, 11]. It may be that other cells lack the appropriate oxygen sensing pathway, or that some additional channel subunit is required, or that uncontrolled over expression of these channels dilutes/titrates out some other important co-factor. Whatever the reason for this failure, it has been a major impediment to the investigation of putative oxygen sensing pathways regulating these channels. Studies conducted in type 1 cells have however provided us with some interesting insights.

The first, and most important, observation is that oxygen sensing is not seen in excised patch recordings [17]. Nor do we see hypoxic inhibition of background K^+^ current in conventional whole cell recordings. Indeed, these experimental techniques result in very rapid and extensive rundown in K^+^ current/channel activity (an approximate 90 % reduction within 10–20 s) [137]. This demonstrates that cellular constituents are essential to maintain normal levels of channel activity under normoxic conditions and to confer oxygen sensitivity on the channel. Thus, although often colloquially referred to as an “oxygen-sensitive” ion channel, there is no evidence yet that TASK channels are directly oxygen sensitive.

One of the cellular constituents that is likely to play a key role in maintaining TASK channel activity is MgATP. Studies using inside-out patches have shown that, post rundown, 5 mM MgATP can increase channel activity by around fivefold [132]. Whilst this is not sufficient to fully restore channel activity to the levels seen in cell-attached patches, it is nonetheless likely to be a highly significant influence on channel activity. This effect requires the magnesium chelated form of ATP and can be mimicked by other Mg nucleotides including MgADP and MgGTP but requires millimolar levels of nucleotides, so that significant activation by MgADP or MgGTP is not anticipated in the intact cell. The *K*_*m*_ for channel activation by MgATP, 2.3 mM, on the other hand, is within the normal physiological range for cytoplasmic MgATP concentration [132]. This observation is of particular relevance to one of the oldest theories of chemoreception, the metabolic hypothesis.

## Mechanisms of oxygen sensing in TASK channels: the metabolic hypothesis

**Fig. 2:**
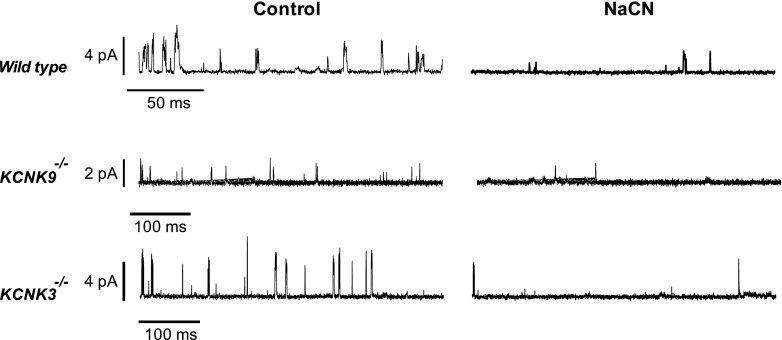
Metabolic regulation of TASK channels. Effects of cyanide (2 mM) on TASK channel activity in cell-attached patches from mouse type 1 cells. *Wild-type* cells show primarily TASK1/TASK3 heterodimer channel activity. *KCNK9* ^*−/−*^ cells (*Task3* KO) show TASK1-like activity. *KCNK3* ^*−/−*^ cells (*Task1* KO) show TASK3-like activity. Note that all forms of TASK channel activity are suppressed by cyanide. Similar effects were also seen for hypoxia and the mitochondrial uncoupler FCCP, see [129]

The metabolic hypothesis posits that oxygen is sensed through changes in mitochondrial energy metabolism. This is supported by a number of key observations. The first is that inhibitors of mitochondrial respiration are all potent chemostimulants [3, 48, 87, 117]. The second is that the oxygen sensitivity of mitochondrial function in type 1 cells appears to be specifically adapted to the role of oxygen sensing [33, 34, 83, 84]. This latter observation has been particularly contentious over the years, but a recent study confirms that the apparent *K*_*m*_ for oxygen utilisation by cytochrome oxidase is extraordinarily high in these cells, possibly uniquely so [12]. Early observations that mitochondrial inhibitors excite chemosensory nerves and increase ventilation have proven remarkably robust and are supported by more recent studies, demonstrating that a wide variety of metabolic inhibitors depolarise type 1 cells and evoke a rise in intracellular calcium [16, 140]. For all inhibitors studied thus far, excitation results from the inhibition of background/TASK channel current. Studies in TASK KO mice have further shown that metabolic inhibitors modulate all three forms of TASK channel in these cells (i.e., TASK1, TASK3 and TASK1/TASK3 heterodimers), see Fig. 2 [129]. This data provides compelling evidence that a metabolic sensing/signalling pathway couples to TASK channels in type 1 cells. It has further been observed that the effects of metabolic inhibitors and hypoxia upon background (TASK) K^+^ current are mutually exclusive [140], suggesting that both signalling pathways converge at the level of mitochondrial metabolism.

Thus, the metabolic hypothesis of oxygen sensing is almost complete. Hypoxia inhibits mitochondrial electron transport [12]; this leads to a fall in cytosolic MgATP (which has been indirectly observed using Mg-sensitive dyes, see [132]), and this leads to TASK channel inhibition. Leaving aside the issue of why type 1 cell mitochondria behave thus, there is another, channel centric, conundrum. Whilst MgATP may stimulate the native TASK1/TASK3 heterodimers in type 1 cells, there is no recognisable nucleotide binding site in either channels primary sequence. Moreover, there have been no reports of MgATP sensitivity for cloned TASK1 or TASK3. It may therefore be that another intermediary, possibly an accessory subunit to the channel, is required to confer MgATP sensitivity.

## Mechanisms of oxygen sensing in TASK channels: the AMP kinase hypothesis

The AMP kinase (AMPK) hypothesis of oxygen sensing is, in essence, another variant of the metabolic hypothesis. It proposes that various potassium channels (including TASK channels) are regulated by phosphorylation via AMP-activated kinase. AMPK is a well-known metabolic energy sensor which pays a key role in coordinating metabolism and cellular energy demand in other cell types. The basic model is that when ATP demand begins to exceed supply, there is a net conversion of ATP to ADP, followed by the conversion of ADP to AMP. AMP then activates the kinase by both displacing ATP which is normally bound to the AMP kinase and in the process making AMPK susceptible to activation by phosphorylation via an upstream kinase, e.g. LKB1 (see [47]). Hence, there is an implied failure of type 1 cell metabolism to maintain cellular ATP levels during hypoxia.

Initial evidence for a role of AMPK in oxygen sensing included the observations that 1) AICAR, an activator of AMPK,stimulated both isolated chemoreceptors and type 1 cells; and 2) that compound C, an inhibitor of AMPK, attenuated the excitatory effects of both AICAR and hypoxia [39, 141]. This was followed by conflicting evidence as to the ability of AMPK to phosphorylate and/or inhibit TASK channels. AICAR was reported to inhibitTASK3 but not TASK1 in H293 cells by Dallas et al. [30]. In contrast, Kréneisz et al. reported that AICAR had no effect upon TASK1, TASK3 or the TASK1/3 concatamer in HEK293 [63]. A more recent study has also failed to find any effect of the AMPK activators AICAR or A769662 on either TASK channel activity or Ca^2+^ signalling in isolated rat type 1 cells or any effect of compound C upon TASK channel inhibition by hypoxia [58]. We have obtained similar results in respect of the lack of effects of AICAR and A769662 on Ca^2+^ signalling in type 1 cells (Buckler unpublished). The role of AMPK in regulating TASK channels in the type 1 cell is therefore currently in doubt.

## Mechanisms of oxygen sensing in TASK channels: role of hydrogen sulfide and carbon monoxide

There has recently been much interest in the role of the putative gaseous signalling molecules H_2_S and CO in oxygen sensing both in the carotid body and in vascular smooth muscle. The reader is directed to a number of recent reviews for the relevant background [91, 110]. Suffice to say that the effects of exogenous sulfide have been tested in the carotid body and isolated type 1 cells, and it appears to mimic the effects of hypoxia in all major respects including the depolarisation of type 1 cells through the inhibition of background K^+^ currents and TASK channel activity [9, 105]. The effects of exogenous H_2_S are manifest at micromolar levels, however, which are sufficient to also inhibit mitochondrial metabolism (as measured in the type 1 cell). Moreover, H_2_S had no additional effect upon TASK channel activity in the presence of cyanide [9]. This suggests that modulation of TASK channels by H_2_S is simply another manifestation of metabolic sensing in type 1 cells. A similar argument may be applied to the effects of carbon monoxide. High levels of CO excite the carotid body, depolarise type 1 cells and inhibit the background K^+^ conductance [4]. At these levels, however, CO will also inhibit cytochrome oxidase, suggesting that the actions of CO on background (TASK) current is again most likely to be due to metabolic signalling.

## Mechanisms of oxygen sensing in TASK channels: reactive oxygen species

Reactive oxygen species have also been implicated in acute oxygen sensing, most notably in the pulmonary vasculature (see [123] for a review) which also expresses TASK-like K-currents [90]. Kim et al. [96] have investigated the effects of hydrogen peroxide on TASK1, TASK3 and the TASK1/TASK3 concatamer expressed in HeLa cells, as well as the TASK channels in type 1 cells. Peroxide was found to evoke an irreversible stimulation of all of these channels in the excised patch configuration but only at exceptionally high concentrations (≥16 mM). This suggests that H_2_O_2_ at least is unlikely to directly modulate TASK channels physiologically.

## Neurotransmitter and autocrine regulation of TASK channels in type 1 cells

The carotid body has a rich diversity of neurotransmitters and neurotransmitter receptors which provide for a complex regulation by neurocrine, autocrine and paracrine signalling events involving type 1 cells, nerve endings and type 2 cells. There have, as yet, been relatively few studies of the impact of such signalling pathways on TASK channels in type 1 cells although this would be a prime target for regulating chemoreceptor excitability. Three substances of likely physiological significance have thus far been identified that can regulate the background TASK channels in type 1 cells.

Adenosine is a powerful stimulant of the carotid body [78, 115, 131] and is released in response to hypoxia. Extracellular adenosine can be derived from two sources: (1) the breakdown of ATP that has been released as a neurotransmitter by the type 1 cell and (2) adenosine efflux from the cell via transporters in response to increased adenosine generation following cytosolic ATP breakdown [24]. Adenosine causes a marked increase in intracellular calcium and membrane depolarisation in isolated type 1 cells, an effect that was mimicked by the TASK1 inhibitor anandamide (see below). Prior exposure to anandamide prevented any further effect of adenosine from which it was concluded that the effects of adenosine were likely to be mediated by inhibition of TASK channels [142]. Although no direct studies of the effects of adenosine on TASK channels have yet been reported in the carotid body, the second messenger pathways implicated, activation of A_2A_ receptors G_s_ increase in cAMP and protein kinase A (PKA) activation, have also been implicated in the modulation of TASK current by GABA. In contrast to the proposed effects of adenosine, GABA is reported to activate TASK current in type 1 cells through activation of GABA_B_ receptors G_i_ and inhibition of adenylate cyclase and PKA activity [40].

Serotonin is another neurotransmitter released from type 1 cells [146]. Serotonin is reported to inhibit the resting K-conductance in type 1 cells leading to depolarisation and cell excitation. These effects are thought to be mediated via 5-HT_2_ receptors and activation of another classical signalling pathway protein kinase C [145].

## Pharmacological modulation of TASK channels in type 1 cells

A number of substances have been observed to inhibit TASK channels in the type 1 cell. Of these, anandamide/methanandamide are probably among the more selective. Anandamide was originally reported to inhibit TASK1 in heterologous expression systems [75] but was subsequently found to also inhibit both TASK3 and the TASK1/3 concatamer [56, 133]. It has been observed to inhibit both background currents [138] and TASK channels in type 1 cells [56]. Ruthenium red, an inhibitor of TASK3 (but not of TASK1, TASK1/3 concatamers or TASK1/TASK3 heterodimers) [52, 60] has only a small (20 %) effect upon TASK channel activity in type 1 cells, supporting the view that TASK3 contributes little to the background TASK-like current overall [56]. Other less selective inhibitors of the TASK-like current in type 1 cells include zinc, barium and quinidine [17]. The effects of recently described selective inhibitors of TASK1 and TASK3, e.g. PK-THPP, A1899 and A293 [7, 42, 82], on type 1 cell TASK channels have not yet been reported. Some of these agents (PK-THPP and A1899) have however been found to act as respiratory stimulants [26]. This is consistent with inhibition of type 1 cell TASK channels and peripheral chemoreceptor excitation. Conversely, the widely used respiratory stimulant doxapram, which is known to excite the carotid body [85, 88], has been found to inhibit cloned TASK1, TASK3 and the TASK1/3 heterodimer [27]. It is therefore anticipated that doxapram will also directly excite the type 1 cell through the inhibition of TASK channels although this has yet to be formally demonstrated. There is little other known pharmacology for TASK in type 1 cells save for the observation that it is strongly activated by the gaseous anaesthetic halothane [17] and weakly activated by isoflurane [95]. The effects of these anaesthetics on channel activity mirror their ability to blunt the acute hypoxic ventilatory response and antagonise type 1 cell responses to hypoxia [93, 94]. Anaesthetic activation of TASK channels is thought to involve two regions of the molecule: a potential binding site located in/near the cytoplasmic end of the second transmembrane domain and another region at the beginning of the c-terminal domain, referred to as the halothane response element, which is important in transducing the effects of anaesthetic [2, 124].

## Future perspectives

One of the most pressing problems is in identifying the signalling mechanisms involved in mediating the effects of hypoxia, metabolic inhibition and cytosolic ATP. Is ATP the only signalling molecule involved in oxygen/metabolic signalling? It certainly has a substantive effect upon TASK channel activity in the type 1 cell, but so to do other unknown cytosolic constituents judging by the degree of channel rundown following patch excision. PIP_2_ depletion is a common cause of channel rundown and has been implicated in TASK channel regulation in some cells/expression systems [22, 70] but not others [23, 68]. The effects of PIP_2_ on type 1 cell TASK channels are unknown. Beyond this, it is difficult to know where to start, but metabolic profiling of other tissues subject to hypoxia or mitochondrial poisons may provide clues.

**Fig. 3:**
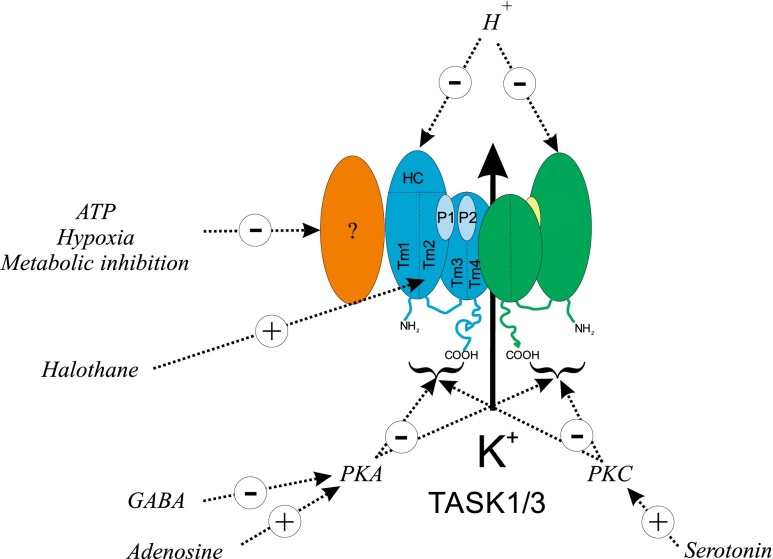
Schematic of TASK channel regulation in type 1 cells of the carotid body. Cartoon depicts key regions of the TASK channels including transmembrane spanning domains Tm1, Tm2, Tm3 and Tm4, pore loops P1 + P2, the extracellular helical cap between Tm1 and P1, and N- and C-terminal domains. Hypoxia/metabolic inhibition/cytosolic ATP signalling is presumed to involve some unknown intermediary, e.g. an accessory subunit (*?*), see text. Modulation by extracellular acidosis probably involves a histidine residue (H98) in the helical cap region (HC). Gaseous inhalational anaesthetics, e.g. halothane, may bind to a region in/near the C-terminal end of the M2 segment [2]. Site of action of the protein kinases PKA and PKC is unknown but is assumed to involve cytoplasmic domains

Another general area of investigation is in the many other ways in which TASK channel activity could be influenced (see Fig. 3). Given their role in setting the type 1 cells resting membrane potential, TASK channels are likely to be highly effective targets for processes regulating the excitability of chemoreceptors overall. This has a number of implications, e.g. (1) short-term neuromodulation of chemoreceptor function by neurotransmitters, (2) long-term plasticity of chemoreceptor responses, (3) carotid body dysfunction (pathology), (4) therapeutic intervention, and (5) unwanted pharmacological side effects.

### Neuromodulation

A role for TASK channels in the neuromodulation of type 1 cell excitability has already been discussed with respect to adenosine, GABA and serotonin. There are however many other important neurotransmitters present in the carotid body including acetylcholine, dopamine and NO [89]. The effects of these pathways on TASK channel activity are all awaiting investigation. It is notable that TASK channels are strongly regulated by G_q_-coupled receptors in other tissues [22, 29, 37, 76, 80, 118, 125], possibly through increase in diacylglycerol [136] and/or by direct interaction with G_q_ α-subunits [23]. Possible direct effects of G_q_ α-subunits and diacylglycerol have not yet been studied in the type 1 cell.

### Plasticity of chemoreceptor responses

There are two physiological processes involving chemoreceptor plasticity. One is in the resetting of chemoreceptor oxygen sensitivity in the early postnatal period. This has been attributed to changes in calcium signalling in type 1 cells [122] and is known to be accompanied by changes in expression patterns and oxygen sensitivity of TASK channels in the type 1 cell [57, 59]. The second form of plasticity is in ventilatory acclimatisation to hypoxia. This is a classical adaptation to life at altitude in which chemoreceptor response to hypoxia is augmented over a period of hours to days [50, 51, 64]. It is likely to be a multifactorial event probably involving short-term plasticity and longer term hyperplasia of type 1 cells which may be driven by oxygen-sensitive gene transcription and/or as a consequence of local signalling [6, 61, 66, 98, 109, 119]. The role of TASK channels in these processes awaits investigation.

### Carotid body dysfunction

A number of clinical conditions have been associated with chemoreceptor dysfunction including sleep apnoea, sympathetic over activity in heart failure and hypertension [1, 77, 99, 100, 111, 116]. These are all complex phenomena in which the role of the carotid body is still at an early stage of investigation. The availability of drugs or other interventions that could modulate TASK channel activity (and thus chemoreceptor excitability) could prove to be of particular value in evaluating the role of chemoreceptor excitation in these situations and, possibly in the long term, in their treatment.

### Pharmacological side effects

Depression of ventilation and inhibition of the ventilatory response to hypoxia is a well-known side effect of a number of general anaesthetic agents which can result in hypoxemia, a significant cause of postoperative morbidity and mortality [44, 62, 134]. A better understanding of how such agents work, particularly their effects on TASK channels, could lead to improvements in anaesthetic design. Moreover, the development of TASK channel inhibitors may provide for a new generation of respiratory stimulants [26] to combat the effects of anaesthetic induced respiratory depression. In summary, there is still a great deal to be learnt about the functional role and regulation of TASK channels in arterial chemoreceptors and their potential value as targets for therapeutic intervention.
